# Is martial arts participation associated with sleep quality among Chinese adolescents? A cross-sectional baseline survey

**DOI:** 10.3389/fmed.2026.1898748

**Published:** 2026-07-20

**Authors:** Minqing Yang, Xiangshan Ge, Defa Zhang

**Affiliations:** 1 School of Physical Education, Suihua University, Suihua, China; 2 School of Foreign Studies, Suzhou University, Suzhou, China

**Keywords:** activity type, adolescents, cross-sectional study, martial arts, organizational form, sleep quality

## Abstract

**Objective:**

This study examined the association between martial arts participation and sleep quality among Chinese adolescents while considering demographic and participation-context factors.

**Methods:**

This study used a cross-sectional design. Data were collected from March 2025 to June 2025 in primary and secondary schools in Guangdong, Jiangsu, Henan, Sichuan, Anhui, and Hainan provinces, China. A total of 6,642 valid questionnaires were included. Martial arts participation and sleep quality were measured using self-report questionnaires. In this study, organizational form referred to the setting in which students participated in martial arts, such as physical education classes, school clubs, after-school training, or self-organized practice. Activity type referred to the specific martial arts or combat-sport category practiced, such as Wushu routines, Sanda, Tai Chi, Taekwondo, or Karate. Multiple linear regression models were used to examine the association between martial arts participation and sleep quality.

**Results:**

The common method bias test showed that the first unrotated factor explained 23.06% of the total variance. This value was below the 40% threshold. Martial arts participation was significantly correlated with sleep quality (*r* = 0.162, *p* < 0.001). After controlling for sex, grade, age, place of residence, and parental education, higher martial arts participation was significantly associated with higher reverse-coded sleep-quality scores, indicating better sleep quality (*B* = 0.005, 95% CI: 0.003–0.007, β = 0.179, *p* < 0.001). Activity type was also significantly associated with sleep quality (*B* = 0.098, 95% CI: 0.055–0.141, β = 0.358, *p* < 0.001).

**Conclusion:**

Higher martial arts participation was significantly associated with better sleep quality among Chinese adolescents. The findings suggest that martial arts may be a potentially relevant school-based activity for adolescent sleep health, although longitudinal or intervention studies are needed to confirm the direction and causality of this association.

## 1 Introduction

Sleep quality is an important indicator of adolescent health and academic adaptation. During adolescence, sleep can be affected by circadian rhythm changes, academic pressure, and screen exposure. These factors may lead to difficulty falling asleep, short sleep duration, and reduced sleep efficiency. Poor sleep is also associated with emotional distress, behavioral problems, and health risks (1–3). Therefore, it is important to identify modifiable lifestyle factors associated with sleep quality. Such factors may provide useful targets for school and public health practice.

Physical activity is widely viewed as an important lifestyle factor for sleep health. The World Health Organization emphasizes the benefits of regular moderate-to-vigorous physical activity (4, 5). It also warns against the health risks of sedentary behavior and excessive screen time. Previous studies suggest that physical activity may be linked to sleep through several pathways, including emotional health, stress responses, and physical recovery (6). In China, schools have been encouraged to provide more time and support for student physical activity (7–9). Against this background, it is useful to examine specific sports. Examining the association between martial arts participation and sleep quality may provide more practical evidence for school health research and program planning.

Martial arts differ from many other forms of physical activity because they combine physical training with breathing control, attention regulation, and emotional adjustment. These features are closely related to possible pathways linking exercise with sleep quality. However, most studies on adolescent physical activity have focused on total activity volume or guideline adherence (10–12). Evidence on the relationship between martial arts participation and sleep quality remains limited. Although martial arts are organized and scalable in school physical education and extracurricular training, their potential sleep benefits have not been well described in large-sample cross-sectional studies. Therefore, it is necessary to examine the independent association between martial arts participation and sleep quality after controlling for demographic and school-context factors (13). These gaps form the direct rationale for this study.

Based on these considerations, this study investigated the relationship between martial arts participation and sleep quality among Chinese adolescents while controlling for demographic and school-context factors. The findings may provide practical evidence for the design and improvement of school martial arts programs (14).

### 1.1 The association between martial arts and sleep health

Sleep quality is a core indicator of adolescent health and development, relating not only to daytime alertness, emotional stability, and academic efficiency but also to obesity, cardiometabolic risks, and mental health outcomes (15). In public health research, physical activity is regarded as a key lifestyle variable influencing sleep. The World Health Organization's guidelines on physical activity and sedentary behavior indicate that regular physical activity is associated with multidimensional health benefits, whereas sedentary behavior and excessive screen time are linked to adverse health consequences, providing a macro-level normative basis for “exercise-sleep” research (4). From a general evidence perspective, adolescents with higher levels of physical activity typically report better subjective sleep experiences, fewer sleep problems, and higher health-related quality of life; conversely, insufficient physical activity and accumulated sedentary time often co-occur with sleep problems (7).

Mechanistic explanations further enhance the interpretability of this association. Guideline development and research reviews generally suggest that physical activity may influence sleep architecture and subjective sleep experience by enhancing homeostatic sleep drive, improving circadian synchronization, promoting energy expenditure and thermoregulation, and reducing stress-related physiological arousal (4). For adolescents, exercise may also indirectly improve sleep by ameliorating emotional and mental health, a pathway particularly relevant during periods of high academic pressure and more prevalent negative emotions (16). Therefore, although different studies vary in sleep measurement (subjective scales or objective monitoring) and activity indicators (total volume, intensity, or guideline adherence), the directional conclusion that “physical activity is associated with better sleep outcomes” is relatively consistent.

In the Chinese context, this transferable evidence has strong practical value. In recent years, the national level has continuously strengthened institutional provisions for school physical activity and health governance, emphasizing time guarantees for physical activity among primary and secondary school students and health promotion objectives, thereby creating policy and organizational conditions for exercise interventions in school settings (9). At the same time, large-scale cross-sectional studies have shown that insufficient physical activity among Chinese primary and secondary school students exhibits significant demographic and school-context differences and is associated with multidimensional health indicators, suggesting that “insufficient exercise” is not merely a behavioral issue but is also shaped by factors such as resources, cognition, and time structure (17). Against this background, moving research from “general physical activity” to “participation in specific sports” and focusing the outcome on sleep quality helps generate evidence at an actionable intervention unit, addressing the practical needs of school sports program selection and implementation optimization.

### 1.2 Psychophysiological rhythm characteristics of martial arts and possible pathways related to sleep

As a traditional Chinese sport, martial arts typically exhibit strong structured training characteristics and a “mind-body unity” practice orientation, which theoretically may exert sport-specific effects on sleep. Compared with sports that are primarily combative or competitive, martial arts training often involves a relatively fixed practice routine, including warm-up, basic skill training, form (taolu) practice or sparring, and cool-down/relaxation phases, making training load and recovery more easily regularized in time. This regularity may align with the circadian rhythm management of sleep, especially when adolescent routines are easily disrupted by academic demands and screen behaviors; stable exercise scheduling may serve as a “behavioral anchor,” helping individuals establish more consistent sleep and wake times (18).

Furthermore, martial arts require a high degree of motor learning and control, emphasizing respiratory coordination, attentional focus, postural control, and body awareness during training, which objectively approximates a composite model of “exercise load stimulation” and “psychological regulation training.” Existing health promotion frameworks point out that physical activity is important for adolescent mental health and stress adjustment, and stress and negative emotions are proximal factors affecting sleep. Therefore, martial arts participation may be indirectly associated with sleep quality through lower tension arousal, better emotional experience, and greater self-efficacy (19, 20). From an implementation perspective in schools, martial arts are often delivered through curricula, clubs, or after-school services, characterized by institutionalization and accessibility; moreover, its cultural attributes may enhance some students' sense of identification and sustained participation motivation, and sustained participation is more likely to bring stable health benefits (21).

It should be emphasized that the association between martial arts participation and sleep is not necessarily uniformly positive; potential asymmetry may exist. If training is scheduled in the evening with high intensity, it may delay the decline of physiological arousal and thus hinder sleep initiation; if training is highly competition-oriented, it may introduce additional psychological burden, offsetting sleep benefits. This implies that in empirical analysis, one should not make linear inferences based solely on the characteristics of the sport; rather, confounding factors such as grade level, academic pressure, place of residence, and family background should be controlled as much as possible in statistical models, and when conditions permit, exposure details such as training frequency, duration, intensity, and organizational form should be further included to more accurately identify the independent association between “martial arts participation” and “sleep quality” and its boundary conditions (22).

### 1.3 Research gaps and contributions

Although the problem of insufficient physical activity among adolescents globally and in China has received sustained attention, and a large body of evidence has been accumulated through guidelines, surveillance, and research, two gaps directly relevant to this study remain in the existing literature. First, many studies use “total physical activity volume” or “whether meeting guidelines” as core exposure indicators, rarely differentiating contextual differences among various sports; however, such differences may manifest in training rhythm, skill-learning pressure, social interaction, and psychological regulation characteristics, all of which may shape the pathway and strength of the association between exercise and sleep (4, 23). Second, in large-scale school-based studies in China, outcome measures often focus on obesity, vision, physical fitness, or general health indicators, whereas specific evidence on sleep is relatively scarce, despite sleep being a key outcome highly relevant to learning, emotion, and daily functioning, and also being susceptible to modifiable lifestyle factors (24, 25).

For example, in national cross-sectional studies, the emphasis has been on describing the prevalence of “insufficient physical activity” and identifying risk factors such as cognition, resources, and time allocation, thereby providing macro-level evidence for policy formulation (10). However, moving from the “meeting guidelines” requirement at the policy and management level to actionable school-based health promotion practices often requires answering more refined questions: under the same time and resource constraints, which sport and in which organizational format may better improve students' key health outcomes. Sleep quality is one such outcome with high public health significance and educational contextual relevance. At the same time, sleep determinants are multi-source; academic pressure, screen behavior, family support, and school schedules may intertwine with sleep and exercise participation, and without proper control, bias or over-interpretation of the association is likely (26, 27).

Therefore, this study uses a cross-sectional baseline survey as a starting point to empirically examine the sport-specific question: “Is martial arts participation associated with sleep quality among Chinese adolescents?” Through descriptive analyses and stratified comparisons, we present the basic distribution characteristics of martial arts participation and sleep quality, evaluate the statistical association between martial arts participation and sleep quality in multivariate models controlling for key demographic and school-context variables, and explore heterogeneity patterns across different educational stages and residential subgroups. The anticipated contribution of this study is to provide sleep-health evidence closer to “actionable intervention units” for school sports program provision and martial arts promotion, and to establish a comparable baseline for subsequent longitudinal follow-up or intervention trials (28, 29).

## 2 Subjects and methods

### 2.1 Study participants

This study used a cross-sectional design. Baseline questionnaire data were analyzed. Data were collected from March 2025 to June 2025. Ethical approval was obtained from the Ethics Committee of the School of Physical Education, Suihua University. The approval number was SHU-IRB-202-008. Permission was obtained from the participants and their guardians before data collection. Random cluster sampling was used. Schools and classes served as natural cluster units. The survey was conducted in primary and secondary schools in Guangdong, Jiangsu, Henan, Sichuan, Anhui, and Hainan provinces, China. Students completed the questionnaires in class under standardized instructions during the data collection period. A total of 6,642 valid questionnaires were included. The sample included 3,726 boys (56.1%) and 2,916 girls (43.9%). Most participants were aged 16–18 years (63.14%). Place of residence included urban areas (2,562, 38.57%), county towns (2,184, 32.88%), and rural areas (1,896, 28.55%).

### 2.2 Methods

#### 2.2.1 Martial arts participation scale

The independent variable was the level of martial arts participation. Martial arts participation was measured using an adapted version of the Physical Activity Rating Scale-3 (PARS-3) developed by Liang et al. (30). The original PARS-3 assesses physical activity participation mainly through exercise intensity, duration, and frequency. In the present study, the wording referring to general physical activity was revised to refer specifically to martial arts or combat-sport participation, while the core scoring framework of frequency, duration, and intensity was retained.

The questionnaire provided examples of martial arts and combat sports, including Tai Chi, Xingyi, Bagua, Shaolin, Changquan, Sanda, Taekwondo, and Karate, to help participants understand the scope of martial arts participation. The adapted scale included four observed indicators: participation experience, frequency, duration, and self-perceived intensity. Participation experience was coded as 0 = no participation and 1 = participation in martial arts or combat sports during the past 12 months. Frequency assessed the number of times students participated in martial arts or combat sports during the past month and was scored on an ordinal scale from 1 to 5, with higher scores indicating more frequent participation. Duration assessed the average length of each session and was scored from 1 to 5, with higher scores indicating longer participation time. Self-perceived intensity assessed the perceived physical load during participation and was scored from 1 to 5, with higher scores indicating greater intensity.

The final martial arts participation score was constructed by combining the frequency, duration, and intensity indicators, following the PARS-3 framework. Specifically, the participation score was calculated as frequency × duration × intensity. Students without martial arts participation during the past 12 months were assigned a score of 0. Therefore, higher scores represented a higher level of martial arts participation. Organizational form and activity type were recorded as contextual variables and were not included in the composite martial arts participation score.

The adapted martial arts participation scale showed acceptable reliability and validity in the present sample. The Cronbach's alpha coefficient was 0.84, indicating good internal consistency. Construct validity was examined using exploratory factor analysis. The Kaiser–Meyer–Olkin (KMO) value was 0.82, and Bartlett's test of sphericity was significant (*p* < 0.001), suggesting that the data were suitable for factor analysis. The factor loadings of the main items ranged from 0.68 to 0.86, supporting the structural validity of the adapted scale.

Two contextual variables were also recorded. Organizational form described how or where students participated in martial arts. Examples included physical education classes, school clubs, school teams, after-school training, and self-organized practice. Activity type described what kind of martial arts or combat-sport activity students practiced. Examples included Wushu routines, Sanda, Tai Chi, Taekwondo, Karate, and other related activities. Therefore, organizational form reflected the participation setting, whereas activity type reflected the specific practice category.

#### 2.2.2 Sleep quality scale

The dependent variable was sleep quality. Sleep quality was assessed using the Pittsburgh Sleep Quality Index (PSQI) (31), a widely used self-report instrument for evaluating subjective sleep quality. The PSQI contains 19 self-rated items covering seven components: subjective sleep quality, sleep latency, sleep duration, habitual sleep efficiency, sleep disturbances, use of sleep medication, and daytime dysfunction. The global PSQI score ranges from 0 to 21, with higher original scores indicating poorer sleep quality. In the present study, the total score was reverse-coded for ease of interpretation, so that higher scores indicated better sleep quality.

The PSQI has been widely used in adolescent health research and has demonstrated acceptable reliability and validity. In the present sample, the internal consistency of the sleep quality scale was acceptable, with a Cronbach's alpha coefficient of 0.81. Construct validity was examined using exploratory factor analysis. The Kaiser–Meyer–Olkin (KMO) value was 0.85, and Bartlett's test of sphericity was significant (*p* < 0.001), indicating that the data were suitable for factor analysis. The factor loadings of the sleep quality items ranged from 0.62 to 0.85, supporting acceptable construct validity in the present sample.

### 2.3 Data analysis

SPSS 31.0 was used for statistical analysis. Descriptive statistics were first calculated. Means and standard deviations were used for continuous variables. Frequencies and percentages were used for categorical variables.

The reliability and validity of the main measurement instruments were examined before the main analyses. Internal consistency was assessed using Cronbach's alpha coefficient. Construct validity was examined using exploratory factor analysis. The Kaiser–Meyer–Olkin (KMO) value and Bartlett's test of sphericity were used to determine whether the data were suitable for factor analysis.

Pearson correlation analysis was then performed to examine bivariate associations among the main variables. Multiple linear regression was used to test the association between martial arts participation and sleep quality. Standardized β coefficients were used as effect-size indicators, and 95% confidence intervals were reported for unstandardized regression coefficients. Sex, grade, age, place of residence, and parental education were entered as control variables. The two contextual variables, namely organizational form and activity type, were also included in the model. Given the high correlation between age and grade, collinearity diagnostics, including variance inflation factor (VIF) and tolerance values, were examined. Because the final analytic dataset mainly contained individual-level questionnaire variables and did not retain complete school- and class-level identifiers, multilevel modeling could not be performed. Therefore, the regression results should be interpreted as individual-level associations.

Harman's single-factor test was used to assess common method bias. This test was based on unrotated exploratory factor analysis of the main self-report items (32). The proportion of variance explained by the first factor was used as the criterion. All statistical tests were two-tailed. A value of *p* < 0.05 was considered statistically significant. Exact *p*-values were reported whenever available; values displayed as 0.000 in SPSS output were reported as *p* < 0.001.

## 3 Results and analysis

### 3.1 Common method bias test

The data were collected using self-report questionnaires at one time point. Therefore, Harman's single-factor test was conducted to assess common method bias. The test used unrotated exploratory factor analysis of the main scale items. The first unrotated factor explained 23.06% of the total variance. This value was below the commonly used 40% threshold. These results suggest that common method bias was within an acceptable range.

### 3.2 Basic information of demographic variables

Among the 6,642 valid participants, 3,726 (56.1%) were boys and 2,916 (43.9%) were girls. The highest proportion of educational stages was high school, with 2,976 participants (44.81%) in the third year of high school, followed by second year (1,245, 18.74%) and first year (1,032, 15.54%); junior high and elementary school together accounted for 1,389 participants (20.91%). The majority of participants were aged 16–18 years (4,194, 63.14%). Regarding place of residence, 2,562 (38.57%) lived in urban areas, 2,184 (32.88%) in county towns, and 1,896 (28.55%) in rural areas. Distribution of mother's educational level was as follows: junior high school or below: 2,856 (43.00%); high school/vocational school: 2,013 (30.31%); college degree or above: 1,773 (26.69%).

Table 1 presents the demographic characteristics of the study sample. A total of 6,642 primary and secondary school students were included, with 3,726 boys (56.1%) and 2,916 girls (43.9%), indicating a relatively balanced sex distribution. Regarding grade distribution, high school students constituted the majority, with the highest proportion in the third year of high school (2,976, 44.81%), followed by the second year of high school (14.45%) and the third year of junior high school (13.37%); grades 3–5 of elementary school accounted for 11.83%. The age structure was consistent with the grade distribution, with the sample primarily concentrated in the 16–18 age group (4,194, 63.14%), followed by the 13–15 age group (24.66%) and the 9–12 age group (12.2%). In terms of place of residence, urban participants accounted for 38.57%, county town participants 32.88%, and rural participants 28.55%, covering all three types of areas. Regarding parental education level, “junior high school or below” was the most common category for both parents, accounting for 48.51% of fathers and 55.19% of mothers; high school/vocational school accounted for approximately one-quarter; college/university degrees accounted for about 15%−19%; and postgraduate education or above accounted for about 6% for both parents.

**Table 1:** Demographic characteristics of the participants.

Name	Options	Frequency	Percentage (%)	Cumulative (%)
Gender	Male	3,726	56.10	56.10
	Female	2,916	43.90	100
Grade	Grades 3–5	786	11.83	11.83
	Grade 7 (Junior 1)	90	1.36	13.19
	Grade 8 (Junior 2)	660	9.94	23.13
	Grade 9 (Junior 3)	888	13.37	36.50
	Grade 10 (Senior 1)	282	4.25	40.74
	Grade 11 (Senior 2)	960	14.45	55.19
	Grade 12 (Senior 3)	2,976	44.81	100
Age	9–12 years	810	12.20	12.20
	13–15 years	1,638	24.66	36.86
	16–18 years	4,194	63.14	100
Place of residence	City	2,562	38.57	38.57
	County town	2,184	32.88	71.45
	Rural area	1,896	28.55	100
Father's education	Middle school or below	3,222	48.51	48.51
	High school/technical secondary school	1,782	26.83	75.34
	Associate/Bachelor's degree	1,260	18.97	94.31
	Graduate degree or above	378	5.69	100
Mother's education	Middle school or below	3,666	55.19	55.19
	High school/technical secondary school	1,632	24.57	79.77
	Associate/Bachelor's degree	1,008	15.18	94.94
	Graduate degree or above	336	5.06	100
Total		6,642	100	100

A = martial arts participation; W = sleep quality. ^**^p < 0.01.

### 3.3 Correlation matrix

Table 2 shows that the overall correlations among variables were low to moderate. Grade and age were highly positively correlated (*r* = 0.914, *p* < 0.01), consistent with the logical progression of school levels in the sample and also indicating that collinearity between the two should be considered in subsequent models. Place of residence was significantly positively correlated with parental education level (father: *r* = 0.36, mother: *r* = 0.34, both *p* < 0.01), suggesting that higher levels of urbanization are associated with higher family educational resources. Martial arts participation (A) was significantly positively correlated with sleep quality (W) (*r* = 0.162, *p* < 0.01), indicating a statistical association between the two. Furthermore, sleep quality was negatively correlated with sex, grade, and age, and positively correlated with place of residence and parental education level (all reaching statistical significance).

**Table 2:** Correlation matrix of the main variables.

	Gender	Grade	Age	Place of residence	Father's education	Mother's education	A	W
	1	2	3	4	5	6	7	8
Gender	1							
Grade	0.138^**^	1						
Age	0.136^**^	0.914^**^	1					
Place of residence	−0.052^**^	0.129^**^	0.138^**^	1				
Father's education	0.006	−0.178^**^	−0.176^**^	0.368^**^	1			
Mother's education	0.015	−0.226^**^	−0.220^**^	0.347^**^	0.782^**^	1		
A	0.065^**^	0.173^**^	0.169^**^	0.120^**^	0.085^**^	0.109^**^	1	
W	−0.136^**^	−0.236^**^	−0.210^**^	0.162^**^	0.251^**^	0.284^**^	0.162^**^	1

### 3.4 Regression analysis

Table 3 shows that the overall regression model was significant, F (9, 6632) = 45.385, *p* < 0.001. The model explained 5.8% of the variance in sleep quality (*R*^2^ = 0.058), indicating a small overall effect size and suggesting that the included demographic, family-background, and martial-arts-related variables accounted for only a limited proportion of individual differences in sleep quality. The Durbin–Watson value was 1.891, indicating acceptable residual independence. Although age and grade were highly correlated, the VIF values for grade and age were 2.211 and 2.090, respectively, which were below the commonly used threshold of 5. This suggested that severe multicollinearity was not present in the regression model.

**Table 3:** Multiple linear regression analysis of sleep quality.

	Unstandardized coefficients		Standardized coefficients	*t*	*p*	Collinearity diagnostics		95% CI
	*B*	Sth. error	Beta			VIF	Tolerance	
Constant	1.677	0.082	–	20.441	<0.001	–	–	
Gender	0.06	0.020^**^	0.036	2.932	<0.001	1.053	0.950	0.021–0.099
Grade	0.024	0.012^*^	0.062	2.087	<0.01	2.211	0.452	0.001–0.048
Age	0.060	0.035	0.051	1.726	0.084	2.090	0.478	−0.009 to 0.129
Place of residence	0.081	0.013^**^	0.079	6.095	<0.001	1.187	0.843	0.056–0.106
Father's education	0.027	0.017	0.031	1.570	0.116	2.659	0.376	−0.006 to 0.060
Mother's education	−0.038	0.018^**^	−0.041	−2.104	<0.01	2.702	0.370	−0.073 to −0.003
Martial arts participation	0.005	0.001^**^	0.179	7.618	<0.001	1.183	0.845	0.003–0.007
Organizational form	0.010	0.008	0.016	1.292	0.196	1.031	0.970	−0.006 to 0.026
Activity type	0.098	0.022^**^	0.358	4.397	<0.001	1.237	0.809	0.055–0.141
*R^2^*	0.058							
Adjusted*R*^2^	0.057							
*F*	*F* (9,6632) = 45.385, *p* < 0.001							
D-W value	1.891							

The dependent variable was the reverse-coded PSQI score; therefore, higher scores indicate better sleep quality. *B* represents the unstandardized regression coefficient, β represents the standardized regression coefficient, and CI represents the 95% confidence interval for *B*. Activity type and organizational form were entered according to their coded categories.

After controlling for sex, grade, age, place of residence, and parental education, higher martial arts participation was significantly associated with higher reverse-coded sleep-quality scores, indicating better sleep quality (*B* = 0.005, 95% CI: 0.003–0.007, β = 0.179, *t* = 7.618, *p* < 0.001). Activity type was also significantly associated with sleep quality (*B* = 0.098, 95% CI: 0.055–0.141, β = 0.358, *t* = 4.397, *p* < 0.001). In contrast, organizational form was not significantly associated with sleep quality (*B* = 0.010, 95% CI: −0.006 to 0.026, β = 0.016, *t* = 1.292, *p* = 0.196). These findings suggest that the specific martial arts or combat-sport category practiced may show a stronger statistical association with sleep quality than the broad participation setting. However, given the cross-sectional design and categorical coding of activity type, these associations should be interpreted cautiously and not as causal effects.

## 4 Discussion

### 4.1 Overall association between martial arts participation and sleep quality and its implications

After controlling for demographic and family-background factors, higher martial arts participation remained significantly associated with higher reverse-coded sleep-quality scores, indicating better sleep quality. This finding is consistent with previous guidelines and reviews suggesting that regular physical activity is often linked to better sleep outcomes (33–35). However, because this study used a cross-sectional design, the result should be interpreted as a statistical association rather than evidence of a causal effect. Adolescents are vulnerable to sleep problems. This may be due to circadian rhythm delay, academic pressure, and screen exposure. Therefore, physical activity is an important modifiable lifestyle factor in school health promotion (36).

Previous studies often used total physical activity or guideline adherence as the main exposure. In contrast, this study focused on martial arts participation. This focus is more specific and closer to school practice. When schools provide physical activity programs, they need to decide which activities to offer. They also need to decide how to organize and sustain these activities. Activity-specific evidence can help schools make these decisions. Therefore, this study adds evidence on the link between activity choice and health outcomes (34).

Martial arts participation may be related to sleep through several possible pathways. First, martial arts training usually has a regular structure. It often includes fixed practice components and class schedules. This regularity may act as a behavioral anchor. It may help adolescents develop more stable exercise and sleep rhythms (37). Second, martial arts training emphasizes breathing, attention, and motor control. These features may support emotional regulation. Previous studies suggest that physical activity may be associated with sleep through lower stress and physiological arousal (38, 39). Therefore, the present findings are consistent with these possible mechanisms.

At the same time, caution is warranted in interpreting the magnitude of the present findings. The regression model explained only 5.8% of the variance in sleep quality (*R*^2^ = 0.058), indicating that martial arts participation and the included demographic and family-background variables captured only a small proportion of the determinants of adolescent sleep. Therefore, the statistically significant association between martial arts participation and sleep quality should not be overinterpreted as a strong predictive effect.

Sleep quality among adolescents is shaped by multiple proximal behavioral, psychological, and environmental factors. Screen time may confound the observed association because adolescents with heavy evening screen use may have both less time for organized physical activity and poorer sleep due to delayed bedtime, light exposure, and cognitive arousal. Academic stress may also act as an important confounder, as students with greater academic pressure may reduce extracurricular exercise participation while simultaneously experiencing more difficulty falling asleep or poorer sleep recovery. Sleep hygiene and bedtime regularity are additional factors that may influence both exercise habits and sleep quality. For example, students from families or schools with more stable routines may be more likely to participate regularly in martial arts and also maintain more regular sleep schedules. Mental health status, including anxiety, depressive symptoms, and emotional distress, may further influence both willingness to participate in physical activity and subjective sleep quality (40–42).

These unmeasured factors may partly explain the low explanatory power of the model and may introduce residual confounding or selection effects. Therefore, martial arts participation should be understood as one potentially relevant lifestyle factor rather than a comprehensive explanation for adolescent sleep quality. Future studies should include screen time, academic stress, sleep hygiene, bedtime regularity, and mental health indicators in the same analytical framework. This would allow researchers to test whether these variables confound, mediate, or moderate the association between martial arts participation and sleep quality.

### 4.2 Differential effects of demographic and family socioeconomic factors on sleep quality

The results show significant differences in sleep quality across some demographic variables, suggesting that sleep problems are not uniformly distributed among adolescents. This finding aligns with existing research indicating that “sleep is closely associated with emotional distress, behavioral problems, and health risks, and different groups may exhibit different risk profiles” (43, 44). From the perspective of school health promotion, demographic differences imply that the same intervention may vary in acceptability, adherence, and actual benefits across groups, thus highlighting the practical significance of emphasizing differentiation and precision in the discussion.

The presence of sex differences is consistent with the background of physical and psychological changes during adolescence discussed in the introduction. Existing literature indicates that sleep changes during adolescence are closely linked to emotional problems, and emotional distress and stress experiences may be expressed differently across sexes, with different behavioral consequences, thereby indirectly affecting sleep (45). However, because this study did not directly include variables such as screen exposure, emotional state, or academic pressure, it is more appropriate to propose “possible explanatory pathways” rather than definitive conclusions. For example, sex differences may stem from differences in bedtime routines, evening behaviors, or levels of psychological arousal, all of which need to be examined in future studies with more complete variable sets.

Place of residence showed a stable association with sleep quality, and concurrently exhibited a consistent positive relationship with parental education level, providing statistical support for the importance of contextual factors. This result is consistent with the view presented in the introduction that “resources, cognition, and time structure collectively shape health behaviors and health outcomes, and exhibit demographic and contextual differences” (46). In interpretation, place of residence may affect sleep through multiple pathways: for example, school management and curriculum arrangements, intensity of after-school tutoring, commuting time, noise and crowding in the living environment, community safety, and access to exercise spaces. In other words, the significance of place of residence does not necessarily mean that “urban areas are always better or worse”; rather, it serves as a composite indicator, reminding research and practice to understand sleep within specific life contexts (47).

The results for parental education level suggest a relationship between family socioeconomic background and sleep, but with a differentiated pattern. Existing research often regards parental education as an important indicator of family socioeconomic status, which may indirectly affect adolescent sleep through health literacy, bedtime management, educational expectations, and family support (48). In this study, the correlation between father's and mother's education levels was high, meaning that when multiple closely related indicators are included in the same model, the “independent effects” may be statistically diluted. A more appropriate statement in the discussion is that family socioeconomic factors may affect sleep, but such effects are often realized through family environment and daily management practices. Future studies could consider constructing a composite socioeconomic index (combining parental education, occupation, and family resources) or using structural equation or hierarchical models to reduce the uncertainty caused by overlapping indicators (49).

From a policy perspective, these differential results suggest that sleep health promotion should not rely solely on individual-level behavioral advocacy, but should also integrate family and school support systems. For example, in contexts with relatively fewer resources or more unstable routines, simply recommending “more exercise” may be insufficient to improve sleep; it may need to be combined with sleep hygiene education, screen-use management, psychological support, and optimization of school schedules. This view is consistent with the problem awareness expressed in the introduction regarding “identifying scalable protective factors and translating them into school-based and public health strategies” (50, 51).

### 4.3 Role of school stage and contextual factors and discussion of collinearity

The results suggest that school-stage-related factors have a relatively stable relationship with sleep quality, whereas indicators that highly overlap with age show varying degrees of significance in the model. This phenomenon is typical in adolescent research: age and grade are often highly aligned in the sample, but grade more directly carries educational context information such as academic task structure, examination rhythm, and school schedules. Both existing research and the introduction discussion indicate that the increase in sleep problems after adolescence is often accompanied by rising academic pressure, later bedtimes, and increased evening activities (52, 53). Therefore, from an educational reality perspective, school-stage variables may be closer to the changes in “pressure and time structure” and may better explain sleep differences.

From a methodological standpoint, the high correlation between grade and age introduces collinearity risks, making it difficult for some variables to show independent effects after controlling for others. However, the collinearity diagnostics did not indicate severe multicollinearity, as the VIF values for grade and age were both below the commonly used threshold. Therefore, both variables were retained in the model. The rationale was that age mainly reflects biological and developmental differences, whereas grade reflects school-stage characteristics, academic demands, examination pressure, and school schedules. It should be emphasized that a non-significant finding in a multivariate model does not necessarily mean that the factor is unimportant in reality; rather, it may reflect that its explained variance has been absorbed by highly overlapping indicators. Connecting with the analytical approach proposed in the introduction, future studies could improve clarity through different modeling strategies, such as theoretically prioritizing “school stage” over “age” as a developmental indicator, or using stratified analyses to examine the relationship between martial arts participation and sleep separately for different educational stages, thereby reducing the unstable influence of overlapping variables (54).

Regarding the contextual variables of martial arts participation, this study distinguished between organizational form and activity type. Organizational form refers to the participation setting or delivery mode, such as physical education classes, school clubs, school teams, after-school training, or self-organized practice. Activity type refers to the specific martial arts or combat-sport category practiced, such as Wushu routines, Sanda, Tai Chi, Taekwondo, or Karate.

The results showed that activity type was significantly associated with sleep quality, whereas organizational form was not. This finding suggests that martial arts participation should not be viewed as a homogeneous exposure. Different activity types may involve different training loads, movement rhythms, levels of physical contact, and psychological demands (55). Because activity type was entered as a coded categorical variable, the coefficient should be interpreted as indicating differences across coded activity categories rather than a linear dose–response effect. These differences may partly explain why sleep-quality scores varied across activity types.

The non-significant result for organizational form does not mean that the way martial arts activities are arranged is unimportant. One possible explanation is that the categories of organizational form were broad. For example, the same category, such as school club or after-school training, may still include differences in training frequency, training time of day, coaching style, and training intensity. Future studies should collect more detailed information about training frequency, timing, intensity, coaching arrangement, and years of participation (56). This would help clarify how participation settings and specific activity types are related to adolescent sleep.

From a practical perspective, this part of the discussion can further inform school sports implementation: against the background of policies emphasizing physical activity guarantees, if schools incorporate martial arts into their curriculum or after-school services, they may need to pay simultaneous attention to training timing and intensity management, avoiding scheduling high-arousal training too late in key school stages, so as not to offset potential sleep benefits (57, 58). This “activity provision – implementation details—health outcome” chain is precisely the significance of moving from general activity research to activity-specific evidence.

### 4.4 Limitations and future research directions

First, this study used a cross-sectional design. Therefore, causal conclusions cannot be drawn. The findings only show an association between martial arts participation and sleep quality. Reverse causality may exist. For example, adolescents with better sleep may be more likely to participate in regular exercise. Selection effects may also exist. Families with more stable routines may support both regular exercise and regular sleep. In addition, because participants were recruited through cluster sampling from schools and classes, observations within the same school or class may not have been fully independent. As complete school- and class-level identifiers were not retained in the final analytic dataset, clustering effects could not be modeled directly. This may have affected the estimation of standard errors. Future studies should use multilevel modeling or cluster-robust standard errors when complete cluster information is available. Future studies should also use longitudinal designs, natural experiments, or intervention trials to test the causal pathway (59, 60).

Second, there is room for improvement in variable measurement. Both martial arts participation and sleep quality were measured by self-report questionnaires, which may be subject to recall bias and social desirability effects. Moreover, martial arts activities themselves are heterogeneous, and key information such as training time of day, intensity, frequency, and years of participation remains limitedly captured. Existing research suggests that the association between exercise and sleep may vary by intensity and timing. Future studies could supplement these more refined exposure indicators and, where conditions permit, introduce objective sleep measurement to enhance the validity of results and the ability to explain mechanisms (61).

Third, the explanatory power of the regression model was relatively low. The model explained only 5.8% of the variance in sleep quality, suggesting that many important determinants of adolescent sleep were not included. In particular, screen time, academic stress, sleep hygiene, bedtime regularity, and mental health were not directly measured in this study. These variables may operate as potential confounders. For example, excessive screen time may reduce opportunities for physical activity and delay sleep onset; academic stress may limit participation in extracurricular activities and increase physiological or psychological arousal before bedtime; poor sleep hygiene and irregular bedtime routines may reflect broader family and school management patterns that are also related to exercise participation; and mental health problems may influence both motivation to participate in martial arts and perceived sleep quality. Existing literature also emphasizes that screen exposure, sedentary behavior, academic pressure, and mental health are closely related to sleep (62, 63). Therefore, residual confounding cannot be ruled out. Future research should systematically incorporate these proximal behavioral and psychological variables and examine their potential confounding, mediating, or moderating effects.

## 5 Conclusion

This study reached three main conclusions. First, higher martial arts participation was significantly associated with higher reverse-coded sleep-quality scores among Chinese adolescents, indicating better sleep quality. This association remained after controlling for sex, school stage, age, place of residence, and parental education. Second, sleep quality differed across demographic and contextual groups. School stage, place of residence, and family socioeconomic characteristics were related to sleep quality. Third, the association between martial arts participation and sleep quality may vary by participation-related characteristics. In particular, activity type was significantly associated with sleep quality, whereas organizational form was not significantly associated with sleep quality. These findings suggest that both the content and implementation details of martial arts programs should be considered when discussing the relevance of martial arts to adolescent sleep health. These findings suggest that the content and implementation details of martial arts programs should be considered when discussing the relevance of martial arts to adolescent sleep health.

## Data Availability

The original contributions presented in the research have been included in the article/supplementary materials. For further inquiries, please contact the corresponding author.
